# The design and development of a stented tissue mitral and aortic heart valve replacement for human implantation

**DOI:** 10.5830/CVJA-2010-065

**Published:** 2012-04

**Authors:** Murray Legg, Edward Mathews, Ruaan Pelzer

**Affiliations:** Centre for Research and Continued Engineering Development, North West University, Pretoria, South Africa and consultant to TEMM International (Pty) Ltd; Centre for Research and Continued Engineering Development, North West University, Pretoria, South Africa and consultant to TEMM International (Pty) Ltd; Centre for Research and Continued Engineering Development, North West University, Pretoria, South Africa and consultant to TEMM International (Pty) Ltd

**Keywords:** aortic valve replacement, mitral valve replacement, tissue valve

## Abstract

**Abstract:**

A study was conducted into the development of a mitral and aortic heart valve replacement that caters for patients having suffered valve damage due to stenosis or rheumatic fever. The appeal of the valve is that it is constituted from a solid frame housing pericardial tissue leaflets, and allows the patient freedom from post-operative blood-thinning medication. The valve is designed to appeal to patients in developing areas of the world, as it features a clip-in mechanism to secure the valve assembly into the sewing ring, which is stitched in independently of the frame and leaflets. Re-operative valve replacement would then be made possible when the pericardial leaflets began to calcify. Novel aspects of the design added value to the science of heart valve replacements, through the use of sintered chrome cobalt in the valve components, the insights gained into mechanical testing of pericardium, and the patient benefits offered by the complete design. Further work is planned to fatigue test the assembly, undergo animal trials and make the valve available for commercial use.

Two main but divergent solutions address heart valve replacements, namely bioprosthetic types and mechanical valve replacements. Rapid advances in the chemical treatment of tissue valves by glutaraldehyde fixation^1^ and the progression from ball-and-cage valves^2^ to tilting disc^3^ and bi-leaflet^4^ designs were all limited to a few exciting, pioneering years.

Current valve designs, particularly those of the mechanical form, create turbulent blood flow. This turbulence leads to the formation of blood thromboses and haemolysis and ultimately a thrombo-embolic event. Blood-thinning medication must be prescribed on an ongoing basis. This is a major logistical and financial limitation to patients in outlying and impoverished areas.

Tissue valves do not present the risk of stroke to the patient, but are constructed from porcine or bovine pericardial tissue. The body’s immune response to this generally causes calcification and tissue hardening, ultimately detracting from the ideal functioning of the heart valve replacement and it requires re-operative surgery within a 10- to 15-year period.

It has been estimated that worldwide, around 380 000 valve replacements take place annually.^5^ The majority of these patients are elderly and from first-world countries. Developing countries, however, actually face a greater need for valve replacements due to high instances of rheumatic fever. It is purely a socio-economic limitation that prevents valve surgery being more commonplace in these developing countries.

Long-term follow-up studies focusing largely on elderly recipients may skew the opinions on favourable valve-replacement methodologies because minimal emphasis is given to patients’ long-term follow up in developing countries. Unger and Ghosh^5^ discuss this, noting that 85% of all open-heart procedures are performed in countries representing 11% of the current world population. Lack of education and the distances from healthcare facilities may still impede evasion of post-operative complications associated with anti-coagulation. Therefore, there is a need for a valve replacement that requires little or no ongoing supportive treatment.

## Heart valve replacements

Two main failure modes are experienced in natural mitral and aortic valves, namely stenosis and regurgitation. These can occur in isolation or together at both valve positions. Mitral stenosis occurs where the mitral valve obstructs flow from the left atrium into the left ventricle. Mitral regurgitation occurs when the mitral valve allows reverse flow of blood to the left atrium, rather than it all being pumped during systole through the aortic valve into the aorta.

Developing countries, because of infrastructural limitations in the use of antibiotics, generally have high instances of aortic regurgitation induced by rheumatic fever. Such occurrences in the developed world are typically limited to the elderly who contracted rheumatic fever in their youth but were not administered antibiotics.

## Mechanical valves

The largest load placed on mechanical valves is that of transvalvular pressure, which occurs just after valve closure. This causes two types of wear stress, namely impact- and friction-wear stress. Impact-wear stress occurs between the valve occluder and ring, while friction-wear stress occurs between the occluder and strut pivots. The catastrophic failure of the Bjork-Shiley valve^6^ was due to a transient occlusion impact of the disc onto the outlet strut, causing bending stresses in excess of its elastic material limit.

Material fatigue is another area of concern in mechanical heart valve replacements. To this end, the majority of valve manufacturers make use of either a silicon carbon blend, or pure pyrolytic carbon for the formation of their leaflets. The housing and struts are required to be manufactured from particularly strong metals such as titanium.

Cavitation is another significant mode of failure in mechanical heart valves. It is caused by a localised and significant drop in pressure, equal to or below that of the blood vapour pressure, causing the blood to vaporise and form bubbles in the bloodstream. In mechanical heart valves, the sudden closing of the tilting leaflet causes localised high-pressure jets of blood to squeeze past the closing boundary and form vortices and cavitation. Pitting and surface erosion from this cavitation were found on failed Duromedics valves.

## Tissue valves

Development in pursuit of a solution not incurring the limitations that are placed on mechanical valves followed in the form of tissue valve replacements. Bioprosthetic valves have the major advantage of not being susceptible to the thrombo-embolic effects created by the functioning of mechanical valves. Animal tissues, most commonly bovine pericardium or porcine valves are used for these valves. To allow for the facilitation of these tissues to be integrated into a human heart, antigen masking through treatment of glutaraldehyde fixation has been the status quo since the mid 1960s.^7^

Acknowledging at the outset that tissue valves require masking of antigenicity and that the absence of living tissue prevents any possible form of self-repair, the major research on tissue valves has focused on the implications of this parameter.

The treatment of leaflet tissue with glutaraldehyde reduces immunogenicity but does not totally eliminate it. This causes the tissue to respond with cellular immune responses as the body acts to reject the tissue. Studies indicate that the level of cross-linking of the collagen fibres has the effect of reducing the antigenicity.^8^ However, the major challenge facing tissue valve use is still the link between immune response and calcific degeneration, and hence to be able to trace poorly masked immunogenicity.^9^

Inflammatory degradation causes explanted valves to typically be covered in inflammatory cells, or be penetrated by giant cells and macrophages.^10^ Bovine pericardium heart valve prostheses often have macrophages invade the prosthetic collagen, and in porcine valves, as many as 82% of failed valves showed signs of collagen phagocytosis.^11^

Mechanical leaflet damage is a concern in tissue valves, as the use of glutaraldehyde mitigates any option of self-repair mechanisms. Valves in the mitral position are more stressed than those in the aortic position. Studies by Maxwell *et al*.^12^ indicated that up to 75% of failed porcine valves showed a rupture of a free cusp edge.

In a study by Guangqiang *et al*.,^13^ a comparison is drawn between the aortic valve repair (AVR) of 518 patients using Carpentier Edwards (CE) porcine valves between 1974 and 1996 and the AVR with CE pericardial valves from 1991 to 2002. Ten-year survival rates for the porcine and pericardial valves were 34 ± 2 and 38 ± 6%, respectively. Adverse cardiac events of thrombo-embolism (20 ± 2 and 13 ± 2%, respectively) and endocarditis (2 ± 1 and 1 ± 1%, respectively) over a 10-year span were limited and similar for both. Interestingly, the 10-year follow up requiring no re-operation was lower for porcine (90 ± 2%) than for pericardial (97 ± 1%) valves. Major reasons for explant included structural valve degeneration (SVD), endocarditis and periprosthetic leak. The durability of the pericardial valve offered is superior to the traditional CE porcine valve. The pericardial valve’s freedom from SVD and re-operation makes it a favourable bioprosthetic choice for AVRs.

## Bovine pericardium

Fixation with glutaraldehyde was initially considered to greatly reduce the immune response to tissue xenografts. It is now evident, though, that immune responses to glutaraldehyde-fixed pericardium do still occur,^14^,^15^ Antibody generation and immune rejection in bioprosthetic heart valve degeneration is now accepted, but the actual proteins responsible for triggering the immune response are largely unknown. Antigen masking or antigen removal is considered and implemented to improve the durability and lower the body’s rejection response to implanted bioprostheses.

A study was conducted into the material properties of the two types of unfixed bovine pericardium supplied at Glycar Pty (Ltd). These comprised a thin and thick version of unfixed pericardium, forming 16 randomised batches, each batch containing 10 thin and 10 thick sheets. The pericardial sacs used were sourced in Namibia from animals, by circumferential dissection around the top of the heart adjacent to the major vessels. The tissue was in the form of a cone with a spherical tip, obtained from cattle aged between 12 and 36 months.

The mean thicknesses of the thin and thick pericardium were 0.25 ± 0.03 mm and 0.34 ± 0.04 mm, respectively. The tensile strength of the samples was calculated to be 20.75 ± 2.38 and 24.09 ± 3.85 MPa for the thin and thick samples, respectively.

The experimental results indicate that there is an associated increase in tensile strength with an increase in thickness. The increase in strength that the glutaraldehyde provides is a further benefit in support of the use of the thicker version of pericardium, and would ultimately prolong the fatigue life of the leaflets.

The major drawback of using glutaraldehyde-fixed porcine or bovine tissue in prosthetic implantation is calcification.^16^ Although the exact details of the methods with which such calcification occur are not known, it is felt that the aldehyde elements do have an impact on the rate at which the degradation occurs.^17^ In lieu of this drawback, certain pre- and post-processing techniques are implemented to reduce the risk of calcification. These treatments result in the modification of the amino acids, forming a protein mass that is insoluble in the harshest of denaturing environments.^18^ Hardening of the pericardium is prevented by immersing it in distilled water after glutaraldehyde treatment. Any swelling that may then occur is thus catered for once integrated into the frame geometry.

Grabenwöger *et al*.^19^ conducted a study into the denaturing of glutaraldehyde-preserved and dye-medicated photo-oxidised pericardium of rats, by measurement of the growth properties of seeded umbilical vein endothelial cells on biological tissues. Post-processed pericardium treated with L-glutamic acid, which acts to reduce free, unbound aldehyde groups, was then also tested. It was found that severe calcification occurred on the glutaraldehyde-treated pericardium (165 ± 20 mg) but no cell proliferation occurred. Post-fixation-treated pericardium had a greatly reduced development of calcification (89 ± 14 mg) and also suffered no damage. The photo-oxidised tissue had no calcification but extreme cell proliferation did occur.

## Heart valve design

The valve design ultimately converged on a solution by the balancing and contrasting of design considerations and constraints. The physical geometry, the aspects of the valve assembly and the techniques of surgical implantation all share significant importance in the actual functioning of the valve. A significant aspect of the valve design was the decision taken on the method of surgical implantation, and the technique within which the valve should be inserted into the heart. The principles behind this allowed the valve assembly to be clipped into the sewing ring, once the ring had been stitched into place in the heart. Design considerations would stem primarily from these criteria.

The materials used in the valve assembly needed to be biocompatible and reasonably accessible. The design of the valve must take into consideration the potential of mass production and assembly to cater for significant demand. The choice of production method should consider the complexity of the design, and the accuracy and reproducibility within given tolerances within practical time limits and at an acceptable cost. The surgical team’s interaction with the device, including its placement, ease of sizing and means of attachment into the tissue should all be as infallible as possible. These should all ensure that no aspect of the placement or valve functioning could create an opportunity for significant error.

The selection of pericardial tissue and its treatment and fixing is a further design consideration. Also of vital importance is the method of forming the valve leaflets on the frame to the desired shapes and forms, as well as the creation of continuous free leaflet edges.

A number of design iterations were carried out, each version realising shortcomings in the previous design and limitations of the manufacturing process. The first conceptual designs consisted of machined stainless steel bars. The restrictions of having a uniform frame width led to the investigation of manufacturing by means of a wire cutter, and ultimately a chrome cobalt powder sintering machine. This machine is typically used for the accurate manufacture of dental bridges.

**Fig. 1:**
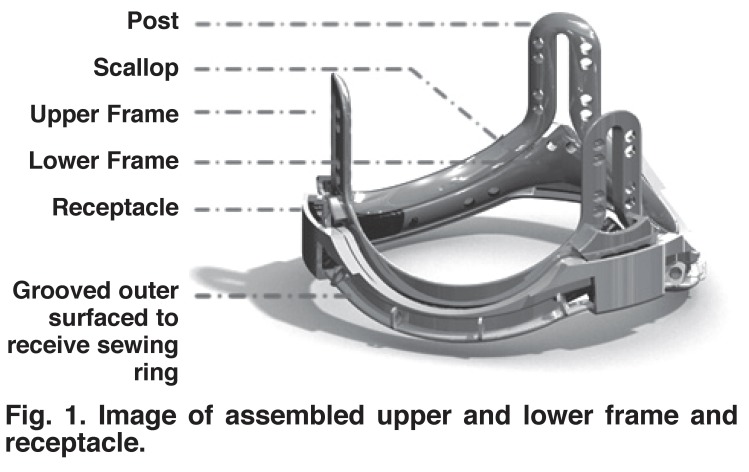
Image of assembled upper and lower frame and receptacle.

The final design provided the best possible solution to the design criteria mentioned. Fig. 1 shows the assembled valve components without the leaflets, comprising the upper and lower components of the leaflet-bearing frame, together with the receptacle, but without the sewing ring in place. Fig. 1 also shows the continuous gap between the upper and lower frame components, and the area where the pericardium meets at the base of the posts in the upper frame. From this base region to the apex of the post, the pericardium leaflets come into surface contact with each other and are held in position by a clip that is located outside the post.

The radial width of the support frame and its receptacle was kept to a minimum of 2.5 mm along the scallops, and 3.0 mm at the posts. The valve frame receptacle is intended for supra-annular insertion. A running 2.0-mm circumferential footprint of the receptacle will lie outside the confines of the native aortic valve annulus. The effective orifice of the valve will therefore be within a 10 to 15% tolerance of the natural orifice of the native valve. A potential disadvantage is that in some patients, the openings to the coronary arteries lie just above the frame annulus, so that the placement of the posts would have to be rotated and positioned accordingly.

**Fig. 2:**
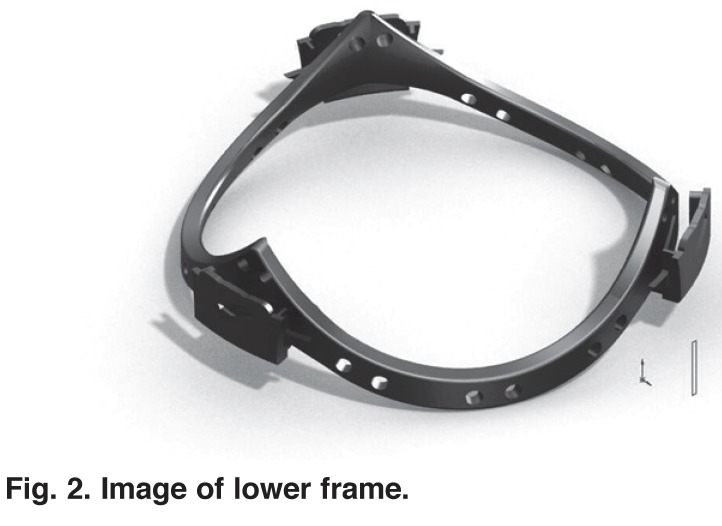
Image of lower frame.

The cross section of the lower frame is hydrodynamically shaped and flared. The surface that opposes a similar-shaped surface on the upper frame to form the gap in which the pericardium is gripped has a width of 1.0 mm and the edges are rounded to avoid the risk of damaging the pericardium as it flexes. The complete bottom frame is shown in Fig. 2. The shape of the lower edge of this component conforms accurately to the shape of the corresponding surface of the upper edge of the lower component, allowing a gap between the components that is inclined at 10 degrees to the horizontal, sloping up toward the centre of the valve.

The gap between the frames, within which the pericardium is held, is designed to be on average 300 μm wide (assuming a pericardium approximately 250 μm thick, with polymer coating of 25-μm thickness on each side of the gap). The pericardial thickness may vary between 200 and 300 μm, and the thickness of the polymer coatings will vary depending on the exact nature of the polymer, its viscosity and drying conditions. Therefore, a locking mechanism has been designed to allow the upper and lower components of the frame to be locked with a gap. This gap takes into account variable pericardial thicknesses, and moreover, can be locked once a predetermined compressive force has been applied to close the gap in which the pericardium is located.

The grooved outer surface of the receptacle has a series of rings that restrain the suture that binds the sewing ring material to the receptacle (see Fig. 1). This material is rolled into a standard-shaped sewing ring for passage of the surgical sutures used to anchor the valve. The flared areas of the receptacle receive the locking mechanism of the upper and lower frames, which engage in a double-clip system at each of the three post regions.

**Fig. 3:**
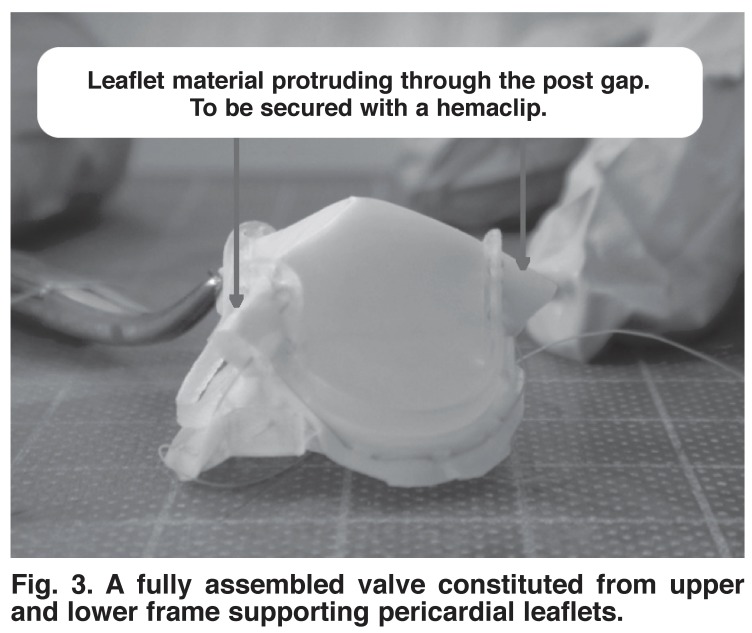
A fully assembled valve constituted from upper and lower frame supporting pericardial leaflets.

The assembly of the valve components, as well as the integration of the pericardial tissue into the frame was undertaken to prove the viability of the concept. The frame components used in this experiment were manufactured from a polymer certified for surgical assistance and suitable for human tissue contact. The assembled pericardial and frame components are shown in Fig. 3.

Chrome cobalt sintering was used for the first time in recorded heart valve literature to manufacture valve stents. Used currently for dental applications, the sintering approach provided an intricately manufactured product, with accurate reproducibility from biostable material at an attractive cost and production time.

The leaflet material protruding through the posts combined to form a tissue thickness of between 0.55 and 0.70 mm. The post, containing a gap of 0.90 mm, allowed for ease of protrusion of this fold of tissue. In order to secure the pericardium in place, a surgical hemaclip, made from approved stainless steel grade 5, is accurately applied after the pericardium has been pulled through the gap. The hemaclip, together with the tissue, form a composite section wider than the post-gap opening, preventing it from pulling though the gap in the posts. The integrity of the hemaclip also supports the vertical aspect of the pericardium, ensuring that it does not fold and collapse under gravity.

## Conclusion

As the world population fast approaches seven billion and the growth rate of developing nations leads the way, the instances of heart valve disease become more prevalent. Currently, the market supplies heart valve replacements largely to those older than 65 years of age and who reside in developed parts of the world. A vast portion of the market is limited by low income and poor access to medical facilities. Lack of financial support prevents any hope of a prolonged life after heart valve replacement.

From criteria pertaining to developing world infrastructure, surgical expertise and patient lifestyle, a valve was designed for use in these countries. In order to ensure a successful final product, the components, materials, assembly and manufacturing techniques were all chosen to be cost effective and accessible. The related surgical procedures are simplified so that the risk of complications is reduced after implantation by surgeons.

Mechanical pericardium tests were performed to understand whether there was an association between thickness and tensile strength. It was found that for unfixed pericardium, tensile strength increased with an increase in tissue thickness. This motivated an appropriate choice of pericardium in the valve assembly, offering sufficient residual tensile strength to successfully undergo the millions of cycles of testing.

Novel heart valve design features, which had not been implemented in valve designs before or patented in any country prior to this study, were integrated into this valve design. These included the use of a mechanical clipping device between the valve assembly and receptacle, which allowed the surgeon to first stitch in the sewing ring and then secure the valve and leaflet assembly in place. This would further permit less-experienced surgeons to perform valve replacements, with a reduction in risk of complications.

Attractive benefits offered in this valve design addressed the issue of re-operation in patients having received a valve implant in their youth. The valve could easily be swapped for new leaflets, clipping back into the receptacle without removing it from the natural valve annulus. This is complemented by the fact that glutaraldehyde-treated pericardium tissue valves give these patients freedom from ongoing anticoagulation medication.

Although the design of the valve addressed the technical aspects of constraint in a theoretical approach, further work would be required to understand the valve functioning in a simulated and fatigue-testing environment. As a result of findings in these areas, a final product could be made available for commercial use.
